# RNase A Does Not Translocate the Alpha-Hemolysin Pore

**DOI:** 10.1371/journal.pone.0088004

**Published:** 2014-02-04

**Authors:** Besnik Krasniqi, Jeremy S. Lee

**Affiliations:** Department of Biochemistry, University of Saskatchewan, Saskatoon, SK, Canada; Université d'Evry val d'Essonne, France

## Abstract

The application of nanopore sensing utilizing the α-hemolysin pore to probe proteins at single-molecule resolution has expanded rapidly. In some studies protein translocation through the α-hemolysin has been reported. However, there is no direct evidence, as yet, that proteins can translocate the α-hemolysin pore. The biggest challenge to obtaining direct evidence is the lack of a highly sensitive assay to detect very low numbers of protein molecules. Furthermore, if an activity based assay is applied then the proteins translocating by unfolding should refold back to an active confirmation for the assay technique to work. To overcome these challenges we selected a model enzyme, ribonuclease A, that readily refolds to an active conformation even after unfolding it with denaturants. In addition we have developed a highly sensitive reverse transcription polymerase chain reaction based activity assay for ribonuclease A. Initially, ribonuclease A, a protein with a positive net charge and dimensions larger than the smallest diameter of the pore, was subjected to nanopore analysis under different experimental conditions. Surprisingly, although the protein was added to the *cis* chamber (grounded) and a positive potential was applied, the interaction of ribonuclease A with α-hemolysin pore induced small and large blockade events in the presence and the absence of a reducing and/or denaturing agent. Upon measuring the zeta potential, it was found that the protein undergoes a charge reversal under the experimental conditions used for nanopore sensing. From the investigation of the effect of voltage on the interaction of ribonuclease A with the α-hemolysin pore, it was impossible to conclude if the events observed were translocations. However, upon testing for ribonuclease A activity on the *trans* chamber it was found that ribonuclease A does not translocate the α-hemolysin pore.

## Introduction

Nanopore-sensing has emerged as a low-cost and label-free technique for studying biomolecules at single-molecule resolution. Initially it was applied to polynucleotides with the goal of achieving DNA sequencing [1]. Recently, nanopore sensing has also been used for other single-molecule level applications such as studying protein folding, protein conformational heterogeneity, enzyme kinetics, intermolecular interactions, to name just a few [2], [3], [4], [5], [6], [7], [8], [9], [10], [11], [12], [13], [14], [15], [16], [17], [18], [19], [20], [21], [22], [23], [24], [25], [26], [27], [28], [29].

Nanopore sensing is achieved by applying a voltage bias via two Ag/AgCl electrodes across a membrane separating two chambers filled with electrolyte solution and then monitoring the ionic current flow through the nanopore embedded in the membrane using the patch-clamp technique. The nanopores can be biological pores (typically extracted from bacteria) or solid state-pores (typically fabricated from silicon material) [30], [31], [32], [33]. The most widely used biological pore is α-hemolysin secreted by *Staphylococcus aureus.* It is secreted as a monomer which oligomerizes upon binding to a lipid bilayer to form a mushroom-shaped heptameric transmembrane pore [34]. The heptameric pore consists of a vestibule with an interior diameter of 36 Å which leads to the stem with a 14 Å constriction between the vestibule and the stem. In the presence of an open pore, there will be a steady flow of ionic current [34]. When a molecule is added to the electrolyte solution it interacts with the pore and reduces the ionic current relative to the open pore current as a result of partially blocking the flow of ions [31].

It has been reported that the interaction of molecules with the α-hemolysin pore causes three general types of events: bumping, translocation, and intercalation [35], [36]. Bumping events (i.e small blockade events) can be distinguished from the translocation and intercalation events (i.e large blockade events) on the basis of blockade amplitude. However, in order to differentiate between translocation and intercalation events a detailed voltage study must be carried out. For an elecrophoretically driven translocation, duration time will be inversely proportional to the applied voltage [11], [35], [36], [37]. In contrast, for an intercalation event the opposite is expected [35], [36].

While this indirect approach might be suitable in proving elecrophoretically driven translocation of single-stranded DNA and peptides, this is not the case with proteins which have more complex structures, larger dimensions than the pore, and generally low net charge density. In addition, for example, the translocation of proteins through solid-state pores has been shown to be a conjoint and competitive action of diffusion, electrophoresis, and electroosmosis [38]. Therefore, a direct approach is needed to determine if a protein translocates the α-hemolysin pore. With single-stranded DNA, direct evidence of translocation through α-hemolysin pore has been obtained by polymerase chain reaction (PCR) amplification of the *trans* side [1]. In addition to DNA, translocation of oligosaccharides was proved by the direct detection of translocated molecules using high-resolution mass spectrometry [39]. However, while there have been reports of protein translocation through the α-hemolysin pore, there is no direct evidence, as yet, that proteins can translocate the α-hemolysin pore [2], [7], [14], [15].

Most reports show that proteins induce large blockade events when interacting with the α-hemolysin pore [4], [5], [7], [40], [41], [42], [43]. We have analyzed eight different proteins (unpublished data) using alpha-hemolysin and all induce large blockade events. At the first glance, the profile of the events observed are similar to those obtained with small peptides and single-stranded DNA or RNA which can be mistakenly identified as translocation events. However, most proteins analyzed with nanopore sensing are larger than the smallest constriction of the α-hemolysin pore [2], [5], [7], [8], [10], [14], [15], [17], [43], [44]. Therefore, in order for proteins to translocate the pore, they must unfold. In the absence of a denaturing agent, it's not clear if the applied electrophoretic force is sufficient in facilitating protein unfolding. Unlike DNA, direct proof of translocation is difficult to obtain with proteins because in a single experiment only 1,000 molecules, perhaps, might be expected to translocate. Hence, a sensitive detection assay is needed in order to provide a definitive answer to the question of protein translocation through the α-hemolysin pore.

**Figure 1:**
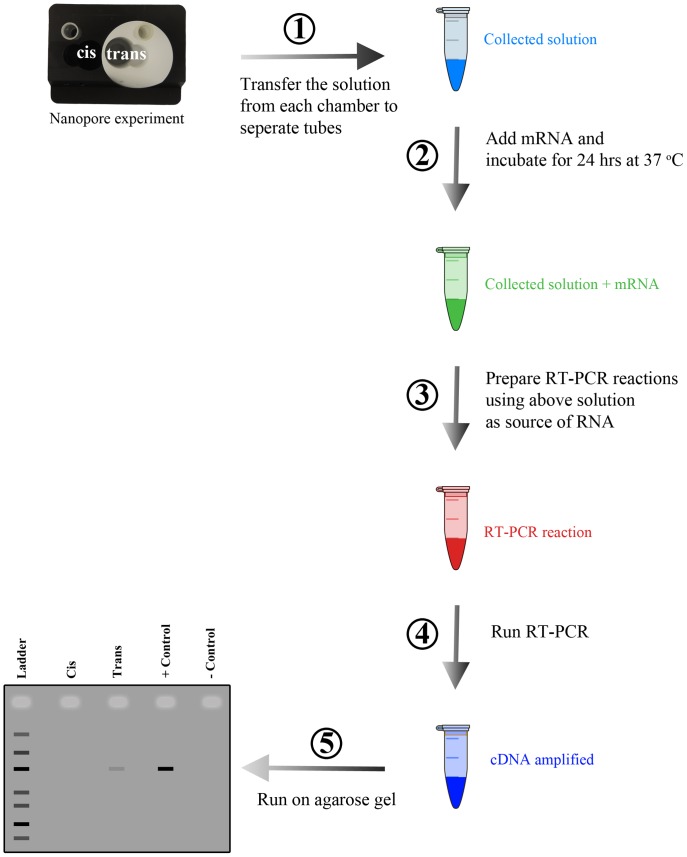
RNase A detection workflow. First, a nanopore experiment is conducted and at the end the solution from each chamber is collected and transferred to a microcentrifuge tube. Second, mRNA is added to the solution collected in step 1 and incubated for 24°C. Third, after incubation the solution from step 2 is used as source of template RNA for RT-PCR reaction. Fourth, RT-PCR is performed. In the fifth step, the end product from RT-PCR is run on an agarose gel. If there is RNase A present in solutions collected in step 1 then there will be a faint band or no band (depending on RNase A quantity) on the agarose gel.

In this study, we take a direct approach to determine if proteins translocate the α-hemolysin pore. In our direct approach an enzyme is subjected to nanopore analysis and then the *trans* side of the pore (i.e opposite from the side where the enzyme is initially added) is tested for enzyme activity. Enzyme activity assays have been previously utilized by other groups to show translocation of proteins through biological channels. For example, Montal and Koriazova used the protease activity of the botulinum neurotoxin light chain to shown that it goes through the neurotoxin heavy chain [45]. An activity based assay has also been used to confirm translocation of proteins through the anthrax toxin pore [46]. For our work we chose ribonuclease A (RNase A) enzyme, a model enzyme, because it is very robust and after unfolding with denaturants and/or disulphide reducing agents it readily refolds to an active conformation [47], [48], [49], [50], [51]. Thus even if it unfolds to translocate the pore, it would readily refold to an active conformation once in the *trans* chamber. RNase A is a 124 amino acid protein with a molecular weight of 13.7 kDa and contains four disulfide bonds [52]. It is positively charged (+4) at physiological pH with a pI of 9.3 [52]. Its dimensions are 3.8×2.8×2.8 nm^3^ [53]. To test for RNase A activity we have developed an assay that relies on a highly sensitive reverse transcription polymerase chain reaction (RT-PCR) technique. The workflow for the RT-PCR based detection assay is shown in Figure 1. Employing the indirect approach (i.e voltage effect on the interaction of the RNase A with the α-hemolysin pore) does not provide a definitive answer to the question of RNase A translocation through the α-hemolysin pore. However, the direct approach utilizing the RT-PCR based detection assay shows that RNase A does not translocate the α-hemolysin pore in the absence of a denaturing agent.

## Experimental Section

### Nanopore Sensing

The 1,2-diphytanoyl-*sn*-glycero-3-phosphocholine in chloroform was purchased from Avanti Polar Lipids Inc. (Alabaster, AL.). Prior to an experiment an aliquot of 1,2-diphytanoyl-*sn*-glycero-3-phosphocholine in chloroform is taken and dried under vacuum to eliminate the chloroform and then re-dissolved in decane at a final concentration of 30 mg/mL. The perfusion unit (cup and holder) was purchased from Warner Instruments (Hamden, CT). The 150 µm aperture found on the wall of the perfusion cup is prepared to accept lipids prior to cup/holder assembly and membrane formation. The preparation is done by pre-coating the aperture (on both sides of the wall) twice with a lipid solution using a paintbrush of size 000. The excess lipid solution on the wall of the cup is dried with nitrogen gas. The perfusion unit is then assembled and the wells in the cup and holder are filled with 1.0 mL of electrolyte solution. The assembled perfusion unit was placed in a copper block which was set on top of an air floating table (Kinnetic Systems, Boston, MA) to shield it from electrical and vibrational interference. The floating table was housed in a Faraday cage (Warner Instruments, Hamden, CT). Finally, the membrane is formed over the 150 µm aperture by applying the lipid solution with 000-sized paintbrush. The multilayer membrane was transformed into a bilayer with repeated brush strokes. The bilayer formation is confirmed through capacitance readings performed by the pClamp 9.0 software (Axon instruments, Sunnyvale, CA). Upon obtaining a stable bilayer, a solution of monomeric α-hemolysin purchased from Sigma-Aldrich (St.Louis, MO), typically 5 µL of 2 µg/mL solution, was added to the *cis* compartment in proximity to the aperture/lipid bilayer. If the first addition of α-hemolysin solution results in no pore insertions, more α-hemolysin solution is added until achieving a stable pore insertion. Following successful pore insertions (eg. 1 to 3 pores), the solution of the molecule to be analyzed was added to the *cis* compartment. All experiments were conducted at a temperature of 22±1°C. A voltage bias of 50 to 150 mV, controlled through the Axopatch 200B patch-clamp amplifier (Axon Instruments) using the voltage clamp recording mode, was applied via two Ag/AgCl electrodes. The electrode in the *cis* chamber was grounded. For all experiments, the signals were low-pass filtered at a cut-off frequency of 10 kHz (100 µs) using the Axopatch 200B amplifier (Axon Instruments) and acquired at 100 kHz (10 µs) frequency using the DigiData 1322A digitizer (Axon Instruments). All events were recorded using Clampex 9 software which is part of the pClamp 9 suite (Axon instruments). The data acquired with Clampex was then analyzed with the Clampfit software, which is also part of the pClamp software suite. Only events with duration times of 50 µs or higher are kept while those with lower duration times are deleted. The blockade amplitudes and duration times obtained with Clampfit are transferred to Origin 7 graphing software (OriginLab Corporation, Northampton, MA). Origin software is used to construct blockade current and time histograms. Each event population on the blockade current histogram is fitted with the Gaussian function to obtain the peak/population blockade current value. The duration time data for each population is plotted separately and the data is fitted with a single exponential decay function.

### Protein Preparation

RNase A was purchased from MP Biomedical with purity of greater than 70% and activity of greater than 70 Kunitz units/mg. The protein was prepared fresh before each experiment at 5 mg/mL in 1 M KCl in 10 mM KPi, pH 7.0, unless stated otherwise. Nanopore experiments were carried out using 30–60 µL of a 5 mg/mL solution. For experiments performed in the absence of guanidine hydrochloride (GdnHCl), the electrolyte used was 1 M KCl in 10 mM KPi, pH 7.0, while in presence of GdnHCl the electrolyte (for *cis* and *trans* chamber) was 1 M GdnHCl, 1 M KCl in 10 mM KPi, pH 7.0. For the analysis of the reduced RNase A, the protein was pre-incubated with a 10 fold excess (per disulphide bond) of tris-2-carboxyethyl-phosphine hydrochloride (TCEP) for 15 minutes before adding to the cup. TCEP was purchased from Sigma Aldrich (Oakville, ON) and was prepared fresh before each experiment to avoid oxidation. In the analysis of purified RNase A, the protein purchased from MP Biomedical was subjected to purification by ion-exchange and size-exclusion chromatography prior to adding it to the *cis* chamber.

For the analysis of completely unfolded RNase A, the protein was prepared in 4 M GdnHCl and 100 mM TCEP at a concentration of 1 mg/mL. The protein was prepared fresh before each experiment and left for incubation overnight at 22±1°C prior to adding it to the cup. The electrolyte used for these experiments was 1 M KCl in 10 mM KPi, pH 7.0.

### RNase A Purification

Gel exclusion chromatography was performed on a 40 cm (32 mL) G-50 Sephadex (GE Healthcare Life Sciences, Baie d’Urfe, QC) column in a buffer of 100 mM KPi, pH 7.0. The column was loaded with 2 mL of 10 mg/mL RNase and 35 fractions of 1 mL were collected. The fractions with the highest absorbance at 280 nm were pooled. The pooled fractions were then dialyzed into 10 mM sodium acetate buffer, pH 5.5. The dialyzed RNase A was further purified by ion exchange chromatography on SP Sepharose Fast Flow (GE Healthcare Life Sciences, Baie d’Urfe, QC), a strong cation exchanger, as per manufacturer's instructions. The salt gradient used was 0–0.4 M KCl. There were 60 fractions of 1 mL collected and the fractions with the highest absorbance at 280 nm were pooled again and used for nanopore experiments. The protein concentration of the pooled fractions was determined by measuring the absorbance at 280 nm and using the molar absorption coefficient (ε) of 9800 M^−1^•cm^−1^ for RNase A [54]. The calculated concentration was 101.5 µM or 1.39 mg/mL. 30–60 uL of this solution was used for nanopore analysis.

### Zeta Potential Measurements

The zeta potential of RNase A was determined in different buffers and pHs with or without KCl. RNase A was prepared fresh at 1.25 mg/mL and the solution was filtered by 200 nm pore size filters (Thermo Fisher Scientific, Ottawa, ON) prior to measuring the zeta potentials. RNase A was prepared in the following solutions: (a) 10 mM KPi (pH 7), (b) 0.1 M KCl in 10 mM KPi (pH 7), (c) 0.1 M KCl in 10 mM KPi, (d) 10 mM TRIS-HCl (pH 8.0), (e) 50 mM KCl in 10 mM TRIS-HCl (pH 8.0), (f) 50 mM KCl in 10 mM Sodium Citrate (pH 4.0), and (g) 50 mM KCl in 10 mM Sodium Carbonate/Biocarbonate (pH 10.0). The zeta potential of each protein was measured with a Zetasizer Nano ZS instrument (Malvern Instruments, Malvern, UK). For all measurements the Huckel approximation (f(Ka) = 1.0) was used. The protein samples were loaded slowly with a syringe into folded capillary cells (Malvern Instruments, Malvern, UK) to avoid formation of air bubbles. The samples were equilibrated for 15 minutes at 25°C before starting the measurements. All measurements were done using the mono-modal measurement mode with a maximum of 100 runs. The voltage was set 50 V for those solutions containing KCl and 150 V for those containing no KCl. This was done to ensure no heating of the sample. The measurement cells were replaced frequently due to the corrosion of the electrodes. Specifically, for those solutions containing KCl a measurement cell was used per single measurement.

### RT-PCR Detection of RNase A

The workflow of the RT-PCR detection of RNase A is shown in Figure 1. Overall the RT-PCR detection of RNase A was divided into two parts: nanopore sensing experiment and RT-PCR based detection assay. For the first part, a nanopore sensing experiment with RNase A was carried out. For these nanopore sensing experiments agarose salt bridges were used. The agarose salt bridges were prepared by filling U-shaped glass tubing with 1.5% nuclease free and PCR pure agarose (VWR International, Edmonton, AB) in 3 M KCl (w/v). The agarose bridges were prepared fresh weekly and stored in 3 M KCl solution. The 3 M KCl solution, the agarose, and the glass tubing used for the salt bridge preparation were all RNase free. The glass tubing was first soaked in RNase Zap solution followed by thoroughly rinsing with RNase free water. The perfusion unit was also soaked in RNase Zap solution followed by thoroughly rinsing with RNase free water and then boiling for couple of hours with RNase free water. RNase A was prepared fresh daily in 1 M KCl in 10 mM KPi (pH 7.4) at 5 mg/mL. The electrolyte used was 1 M KCl in 10 mM KPi (pH 7.4) and was prepared fresh weekly with RNase free chemicals in RNase-free water, not DEPC-treated (Life Technologies, Burlington, ON). After pore insertions and prior to adding any protein, the nanopore sensing experiment was left for incubation for about 1 hr. After 1 hr incubation, 245 µL of the electrolyte solution was collected from each chamber and transferred to non-sticky RNase-free microcentrifuge tubes (Life Technologies, Burlington, ON). These solutions were used as controls to make sure that the apparatus, buffer, α-hemolysin solution, and lipid solution were not contaminated with RNase A. The *cis* and *trans* chambers were then refilled with fresh buffer and 30–60 µL of 5 mg/ml protein solution was added to the *cis* chamber. This was done while the membrane was intact and the desired number of pores were inserted. If the membrane was broken after adding the protein, the experiment was abandoned and a new experiment (with RNase A free apparatus) was restarted. Following, successful completion of the nanopore experiment, 245 µL of the electrolyte solution was collected from each chamber again. These solutions were collected while ensuring the membrane and the α-hemolysin pores remained intact (i.e no RNase a contamination of the *trans* chamber). The solutions were transferred to non-sticky RNase-free microcentrifuge tubes. The non-sticky RNase-free microcentrifuge tubes were used to avoid sticking of RNase A to the walls of the tubes. In addition, RNase free barrier tips with low-binding surface (Ultident Scientific, St. Laurent, QC) were the only type of pipette tips used to avoid loss of RNase A molecules in the process. Without the use of the non-sticky tubes and pipette tips with low-binding surface, the detection assay was not reproducible.

For the second part of the process, 245 µL of RNase free water and 10 µL of 5 ng/mL RNA was added to each of the 4 tubes collected to a final volume of 500 µL. Additionally, there are two more solutions set up containing no RNase A: one of these solutions (i.e positive control for RT-PCR) was 100 pg/mL globin mRNA in 0.5 M KCl, 10 mM KPi (pH 7.4) and the other was the 0.5 M KCl buffer only (i.e negative control for RT-PCR reaction). These reactions were then incubated at 37°C for 24 hrs. The RNA used was rabbit globin messenger RNA (mRNA) purchased from Sigma (Oakville, ON). Globin mRNA was prepared at stock concentration of 20 µg/mL in RNase-free water, not DEPC-treated, and stored at −20°C. Following incubation period, all solutions were used as a source of template RNA for setting up RT-PCR reactions of 50 µL final volume. The RT-PCR was performed using a one-step RT-PCR kit from Qiagen (Mississauga, ON) and were set up as per manufacturer’s instructions. Each RT-PCR reaction, except the negative control, contained 300 fg of globin mRNA. The primers used were specific for the beta-globin cDNA and were designed using the Primer-BLAST tool from NCBI. The designed primers were purchased from Sigma-Genosys (Oakville, ON). The sequences of the primers are shown in Table 1. Small aliquots of working solutions (10 µM) were prepared and stored at −20°C. The thermal cycler conditions were set up as outlined in the RT-PCR kit handbook. Based on the Tm of the primers the annealing temperature was set at 65°C. The number of cycles used for PCR amplification was 34. The end products were run on a 2.5% agarose gel stained with ethidium bromide and visualized using the gel imaging platform, AlphaDigiDoc (Protein Simple, Toronto, ON). The agarose used was PCR quality. The DNA ladder used was purchased from New England Biolabs (Whitby, ON) and was a low molecular weight DNA ladder which included fragments ranging from 25–766 base pairs.

**Table 1:** The primer sequences.

Primer	Sequence
Beta-globin Forward	TGCCCTGTGGGGCAAGGTGAA
Beta-globin Reverse	TAGGCAGCCTGCACCTGAGGA

## Results and Discussion

### Nanopore Analysis of RNase A Under Different Experimental Conditions

**Figure 2:**
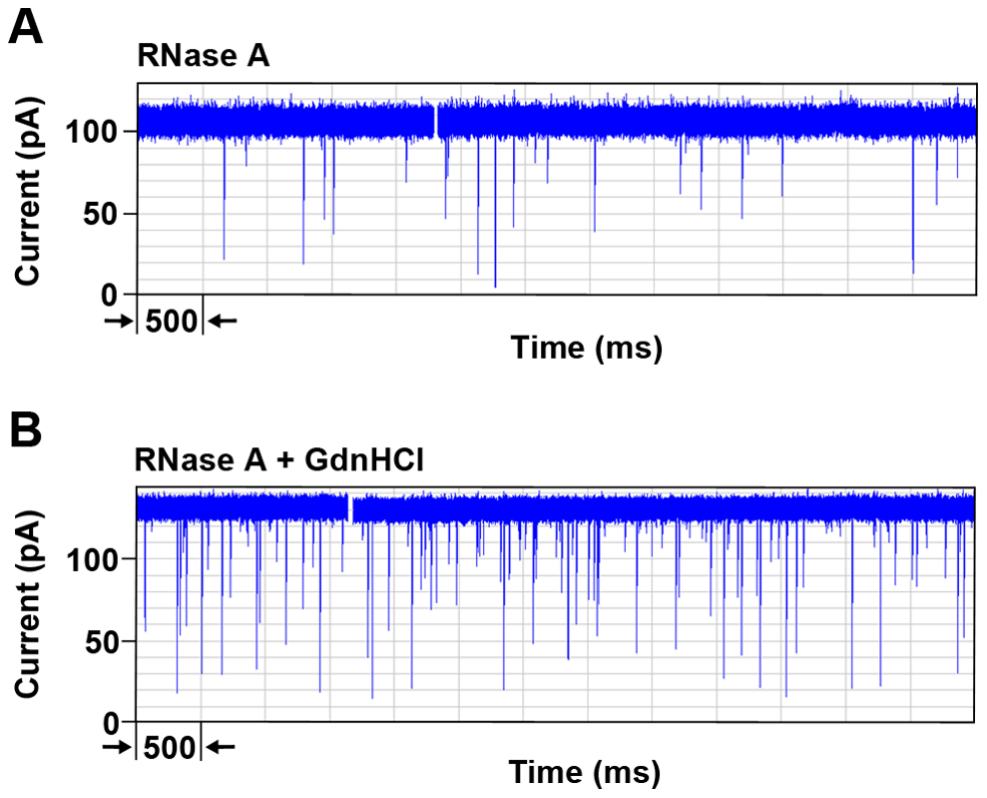
Segments of current traces for the interaction of RNase A with the α-hemolysin pore at 100 mV. In (A) the absence of GdnHCl the frequency of events is lower than (B) in the presence of GdnHCl. The open pore current is higher in presence of GdnHCl as a result of higher conductivity of GdnHCl. Note the increase in frequency of the events and the change in proportion of large blockade events in the presence of GdnHCl.

**Figure 3:**
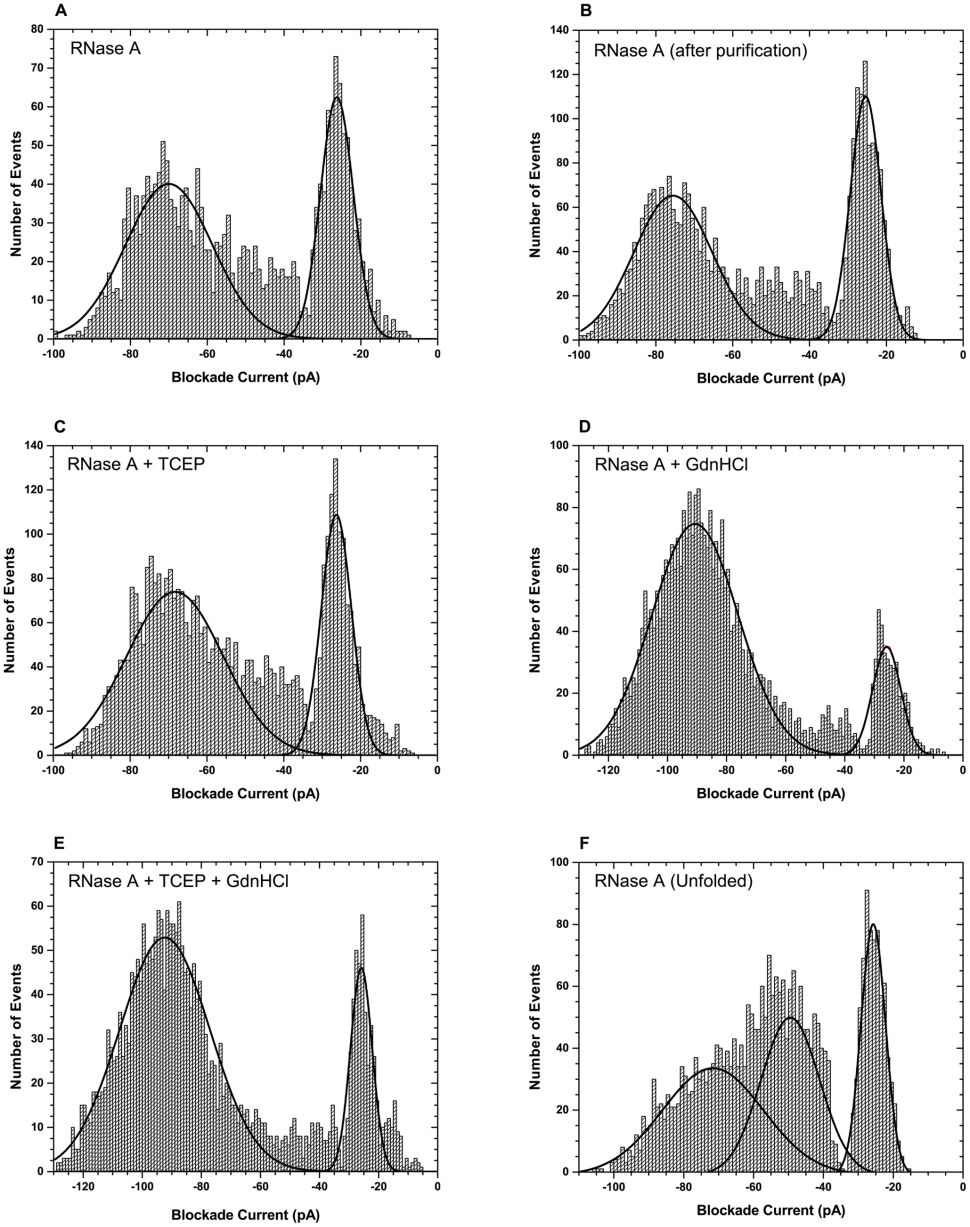
Nanopore analysis of RNase A under different experimental conditions. Blockade current histograms obtained for (A) natively folded RNase A, (B) natively folded RNase A after being subjected to size exclusion and ion exchange chromatography, (C) reduced RNase A, (D) RNase A in presence of 1 M GdnHCl, (E) reduced RNase A in presence of 1 M GdnHCl, and (F) completely unfolded RNase A. For the analysis of completely unfolded RNase A, the protein was pre-incubated in 4 M GdnHCl and 100 mM TCEP prior to adding it to the *cis* chamber. Each event population is fitted with the Gaussian function to obtain the peak/population blockade current value. The peak blockade current values are presented in Table 2. All analysis were performed at 100 mV.

Initial experiments were designed to determine under what conditions RNase A translocates the α-hemolysin pore. Therefore RNase A was subjected to nanopore analysis under different experimental conditions at an applied voltage of 100 mV. First, RNase A was analyzed in the absence of reducing agents and/or denaturing agents. Upon addition of RNase A to the *cis* side (grounded), a significant but unexpected number of events were recorded (Figure 2A). The large number of events was unexpected because the protein has a positive net charge at the buffer pH (pH 7.0) and under the experimental conditions the translocation direction is opposite to the electrophoretic force. Thus the protein would be driven against the electric field. A histogram of blockade currents (Figure 3A) revealed two populations of events, one with large blockade currents (around −70 pA) and the other with small blockade currents (around −26 pA). Over 60% of the events were large blockade events with blockade times of 0.07 ms (Table 2). The large blockade events could be either translocation or intercalation events. However, translocations might be unlikely because RNase A contains four disulfide bonds. To ensure that these events are not a result of impurities, RNase A was then further purified by size exclusion chromatography to remove any small contaminants followed by ion exchange chromatography to remove any contaminants of similar size to RNase A but of different charge. The purified RNase A was then re-examined under the same experimental conditions (Figure 3B). It is clear that the blockade histogram profile still remains the same with 60% of the events being large blockade events (i.e translocation or intercalation events, Table 2). Therefore, the possibility that the events observed with RNase A are due to contaminants is ruled out since even after RNase A was subjected to a second purification by gel exclusion and ion exchange chromatography, there was no significant change in the event frequency and profile (Figure 3).

**Table 2:** Interaction parameters of RNase A with the α-hemolysin pore under various experimental conditions.

Parameter^a^	RNase A	RNase A (purified)	RNase A+TCEP	RNase A+GdnHCl	RNase A+TCEP+GdnHCl	Unfolded RNase A
I_1_ (pA)^b^	−26.3	−25.4	−26.3	−25.8	−25.9	−25.7
I_2_ (pA)	−70.0	−75.5	−68.3	−90.7	−92.4	−49.5
I_3_ (pA)	–	–	–	–	–	−71.6
T_1_ (ms)^c^	0.02	0.05	0.03	0.08	0.04	0.16
T_2_ (ms)	0.07	0.11	0.11	0.14	0.10	0.21
T_3_ (ms)	–	–	–	–	–	0.19
A_1_ (%)^d^	37.2	39.7	31.5	13.28	17.3	23.5
A_2_ (%)	62.8	60.3	68.5	86.72	82.7	35.2
A_3_ (%)	–	–	–	–	–	41.3

a I_1_, I_2_, I_3_, T_1_, T_2_, and T_3_ represent the amplitudes and the durations of the current blockades of the respective event populations presented in Figure 3. A_1_, A_2_, and A_3_ are the percent of total events forming each respective population. The peaks are numbered from right to left. A dash indicates the absence of third event population.

b The error is estimated to be ±1 pA.

c The error is estimated to be ±10%.

d The error is estimated to be ±1%.

With small peptides and polynucleotides the large blockade events would be attributed to translocation of the molecule through the pore. However, native RNase A is larger than the smallest diameter of the pore. Therefore in order for the protein to translocate it would have to unfold. Could the applied electrophoretic force facilitate the unfolding and subsequent translocation of the protein? Considering the presence of the disulfide bonds this is unlikely to happen in the absence of reducing and/or denaturing agent. For this reason, the effect of reducing the disulfide bonds with TCEP (a reducing agent) was examined (Figure 3C).

As shown in Figure 3C there are still two populations of events observed for the reduced protein. Furthermore, both populations have similar blockade amplitudes and proportions as those observed for the native protein (Table 2). In contrast, when examining RNase A in the presence of 1 M GdnHCl (a denaturing agent), the frequency of events is dramatically increased as seen in Figure 2B and the proportion of the large blockade events is also increased from 62% to 87% (Figure 3D and Table 2). Similar results were obtained with reduced RNase A in the presence of GdnHCl (Figure 3E). Furthermore, when comparing the blockade current peaks as a percentage of the open pore current they all remain the same. At this concentration of denaturant the protein is expected to be only partially unfolded [50], [55]. Thus, the entry of a chain of a protein into the pore will be favoured. This in turn will explain the increase in percentage of events with large blockade events, independent of whether the events are intercalation or translocation. Furthermore, the times for the large blockade events are almost similar for purified native RNase A and reduced RNase A in the presence and absence of GdnHCl. Based on these results it can be concluded that TCEP has little or no effect on the interaction of the protein with the pore. On the other hand GdnHCl has a large effect on the frequency of the events and on the proportion of the translocation/intercalation events. Similar effects of GdnHCl have been reported with MBP [2]. However, in the case of MBP the authors reported no events in the absence of denaturant. Overall the results obtained here were unexpected since the protein is much larger than the pore, contains disulfide bonds, and has a positive net charge.

Since the protein could only be partially unfolded in 1 M GdnHCl and partially folded protein might or might not translocate the pore, it is not clear whether the events are translocations. Therefore, it’s important to examine the interaction of completely unfolded RNase A with the α-hemolysin pore and compare the results. RNase A is completely unfolded and reduced in 4 M GdnHCl and 100 mM TCEP [55]. Since α-hemolysin pore cannot withstand these denaturant concentrations, the protein was denatured and reduced outside the cup and then added to the *cis* chamber [2]. Interaction of unfolded RNase A with the α-hemolysin induced three event populations (Figure 3F). There is a clear bumping peak at around −26 pA (far right peak). The other two populations are partially merged together which made it difficult to obtain good Gaussian fits. The middle peak at around −50 pA must be bumping events as well because their current blockade is too small to be an intercalation or translocation event for a protein of this size. On the other hand, the far left peak (around −72 pA) could be translocation events since the protein is fully unfolded before adding to the cup. Interestingly, both folded and unfolded RNase A molecules give events with blockade currents of about 70% current block. In contrast, the blockade times for unfolded RNase A are almost twice as large as those observed for folded (i.e in the absence of a denaturing agent) or partially unfolded RNase A (i.e in the presence of 1 M GdnHCl) (Table 2). Considering that the protein is fully unfolded one would expect it to freely translocate the pore and therefore produce a larger percentage of events with large blockade amplitudes. From the inspection of Figure 3F, this was not the case. In fact, the proportion of the events with large blockade amplitudes was smaller compared to the folded or partially unfolded protein. The simplest explanation for this result is that the large blockade events observed with the completely unfolded protein are indeed due to translocation of the protein through the pore, whereas with the partially unfolded or the native protein the large blockade events are due to intercalation of the protein. This is because the translocation of the fully unfolded protein would not be hindered by the size of the pore, whereas for partially unfolded protein the size of the pore will be a limiting factor. Another explanation for the small number of events with large blockade amplitudes might be that the protein refolds over time after being added to the *cis* chamber, which is expected for RNase A [48], [51], [55].

### Voltage Effect on Interaction of RNase A with the α-hemolysin Pore

The effect of voltage on the translocation parameters of peptides and proteins has been studied previously by our group and others [2], [3], [5], [6], [13], [36], [37]. It's been suggested that the voltage effect on the interaction of a molecule with the pore can be used to provide indirect evidence of protein or peptide translocation through the pore. For example, for an electrophoretically driven translocation, the duration times are expected to be inversely proportional to the applied voltage. On the other hand, the frequency of events is expected to be linearly proportional to the applied voltage [3], [36], [37]. Furthermore, a molecule translocating the pore should induce the same percent current blockade independent of the voltage [6], [11]. This is because the volume occupied by the pore is not dependent on the voltage. If these three conditions are met then one can assume that the molecule has translocated the pore. In the case of an intercalation event, the duration times are expected to increase with the applied voltage [35]. The reason why current measurements provide indirect proof of translocation at best, is because the blockade events observed with proteins could be as results of protein translocation or non-specific protein binding/unbinding to the pore [11]. Therefore, the voltage effect on the interaction of RNase A with the α-hemolysin pore was taken as an indirect approach to determine if the events observed with RNase A are translocations.

**Figure 4:**
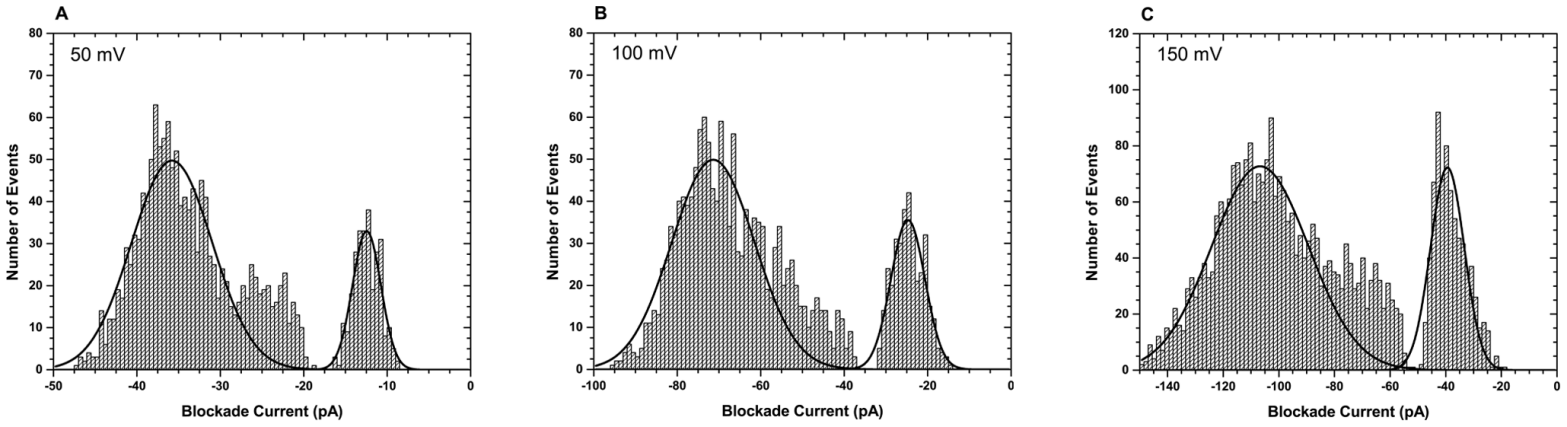
Effect of voltage on the interaction of RNase A with the α-hemolysin pore. Blockade current histograms obtained for RNase A at (A) 50 mV, (B) 100 mV, and (C) 150 mV.

**Figure 5:**
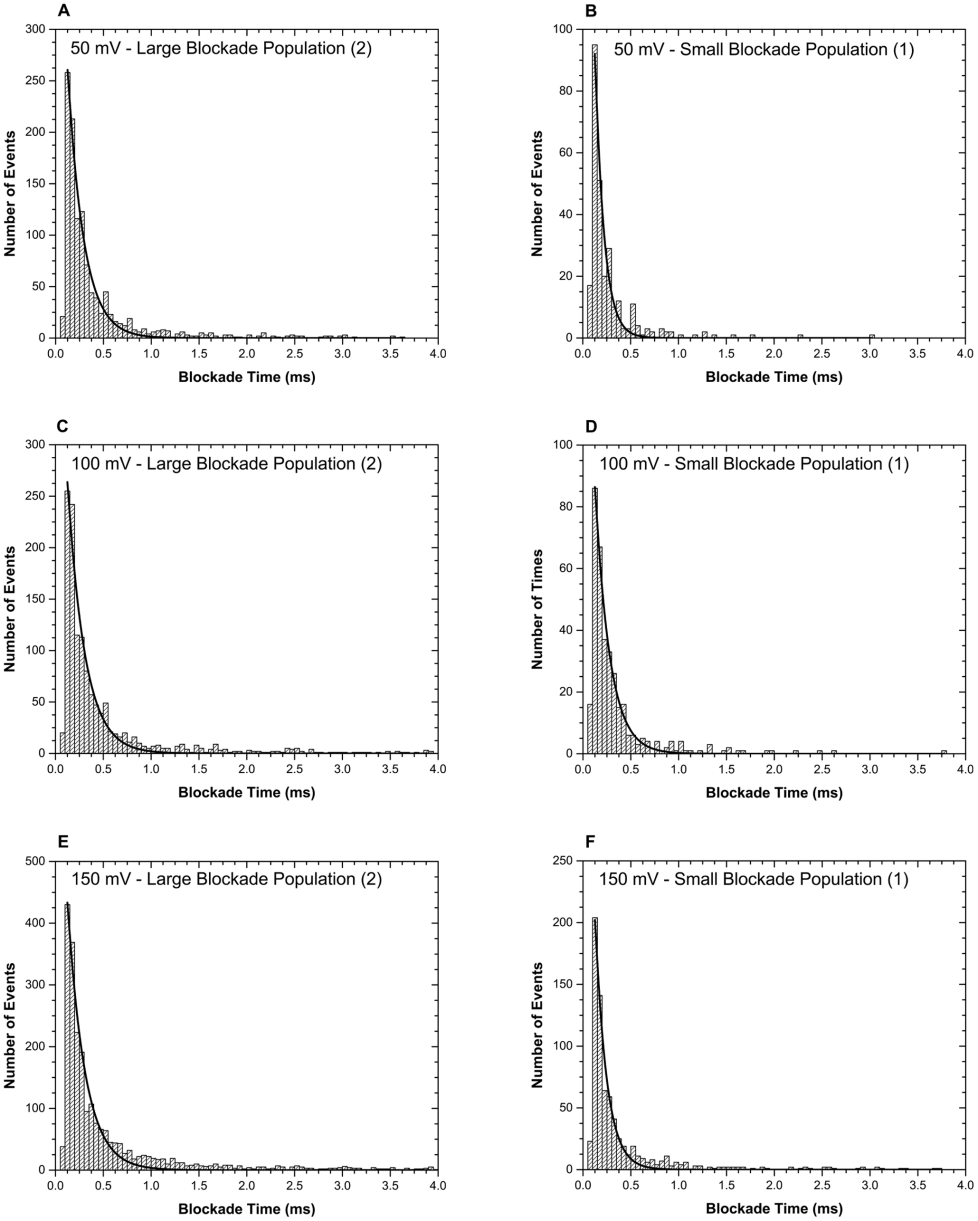
Blockade time histograms for RNase A at 50, 100, and 150 mV. Each individual population of events shown in the current blockade histograms (Figure 4) is fitted with a single exponential decay function to obtain the duration times (dwell times) for each respective population. Panels A and B show the lifetimes of the events forming the large and small blockade populations, respectively, at 50 mV. Panels C and D show the lifetimes of the events forming the large and small blockade populations, respectively, at 100 mV. Panels E and F show the lifetimes of the events forming the large and small blockade populations, respectively, at 150 mV. The duration time values for each voltage are presented in Table 3.

RNase A was subjected to nanopore analysis at 50 mV, 100 mV, and 150 mV and the blockade current histograms obtained for each voltage are shown in Figure 4. As stated earlier, RNase A is positively charged protein (+4) at physiological pH and therefore under the experimental set up used here (buffer pH = 7.0), the protein would have to go against the electric field to translocate the pore (*cis* side grounded). Hence, if the protein is indeed translocating the pore, an increase in the applied voltage should result in an increase in the blockade duration times (i.e dwell times). If the protein is intercalating then the duration times would increase with increased applied voltage. Figure 4 shows the same percent current block (about 71% block) for the translocation/intercalation events independent of the applied voltage. In addition, the frequency of the events increased with voltage. For example, the number of events per pore per minute was 17, 47, and 112 at 50, 100, and 150 mV, respectively. These are two indications of molecule translocation through the pore. However, the duration times for the large blockade events remained unchanged at all three voltages (Figure 5 and Table 3). It was argued that because of low net charge density on RNase A (+0.032) there may be little or no change in event durations as a function of voltage. This hypothesis is intuitively reasonable since higher net charge density would results in higher electrophoretic force acting on the protein. For this reason, calmodulin, MBP, and *E. coli* thioredoxin with different net charge densities were examined at three different voltages (data not shown). Indeed, the change in duration times was the largest for calmodulin which has the largest net charge density while remaining constant for MBP which has similar magnitude of net charge density. The effect of voltage on the interaction of MBP with the α-hemolysin pore has been studied previously and the authors of the study reported an exponential decrease in duration times as a function of the applied voltage [2]. However, in the previous study the analysis was done in the presence of varying concentrations of a denaturant, whereas our analysis was done in the absence of denaturant.

**Table 3:** Effect of voltage on the interaction of RNase A with the α-hemolysin pore.

Parameter^a^	Applied Voltage (mV)		
	50	100	150
I_1_ (pA)^b^	−12.5	−24.7	−39.4
I_2_ (pA)	−35.8	−71.4	−106.7
T_1_ (ms)^c^	0.09	0.15	0.12
T_2_ (ms)	0.17	0.18	0.18
A_1_ (%)^d^	18.9	22.6	25.6
A_2_ (%)	81.1	77.4	74.4

a I_1_, I_2_, T_1_, and T_2_, represent the amplitudes and the durations of the current blockades of the respective event populations presented in Figure 4. A_1_, and A_2_ are the percent of total events forming each respective population. The peaks are numbered from right to left.

b The error is estimated to be ±1 pA.

c The error is estimated to be ±10%.

d The error is estimated to be ±1%.

The lack of change in RNase A duration times with voltage is unexpected. This is because for a translocation or an intercalation event the duration times are dependent on the voltage [36]. Therefore, it is unclear if the events are translocation or intercalation. Furthermore, it is still not clear why there are events observed for RNase A protein even though the protein is going against the electric field. Firnkes *et al.* have shown that translocation of proteins through solid-state pores can still occur even when going against the electric field when the protein is smaller than the pore diameter [38]. They demonstrated that the translocation of proteins through solid-state pores is a conjoint and competitive action of diffusion, electrophoresis, and electroosmosis. Electroosmosis can enhance or counteract electrophoresis depending on the zeta potentials of the protein and the pore. In addition, it was shown that translocation can still occur even when electroosmosis and electrophoresis cancel each other. In such a case the translocation is diffusion controlled, driven by the concentration gradient between the two chambers. However, it should be noted that in their study the size of the pore was not a limiting factor on translocation of the protein. Therefore, the proteins could readily translocate folded. Japrung *et al.* also observed events for a protein which was thought to be going against the electrophoretic flow [56]. However, upon measuring the zeta potential of the protein it was found that the zeta potential of the protein was negative although the protein has a positive net charge, thus indicating charge reversal. The charge reversal was occurring at high salt concentrations of about 200 mM KCl. Hence it was hypothesised that the same might be happening with RNase A.

To investigate this anomalous behaviour with RNase A, zeta potential measurements were conducted in similar solutions to the ones used for nanopore analysis but with lower salt concentrations. This is because the determination of zeta potentials at high salt concentration was impeded by high voltages and currents. Firnkes *et al.* showed that the zeta potential of avidin and streptavidin decreases with increasing salt concentration and eventually reaches a plateau at a concentration above 0.1 mM KCl [38]. Typically, a positive zeta potential is expected at a pH below the pI [38]. However, the zeta potential obtained for RNase A (pI 9.5) in 0.1 M KCl, 10 mM KPi pH 7.0 was −1.62 mV (Table 4). This indicates that RNase A undergoes a charge reversal under the nanopore experimental conditions. Therefore, because of the charge reversal the applied electric field will actually facilitate the translocation of the protein rather than hinder it. In addition, since α-hemolysin pore is slightly anion selective its zeta potential is expected to be very small and negative. Since both the pore and protein have negative zeta potentials, the small electroosmotic flow will counteract the electrophoresis [38]. However, the zeta potential of the pore is expected to be much smaller than that of the protein and thus the translocation direction would be electrophoretic. This explains the large blockade events observed with RNase A even though the protein has a positive net charge. In addition, the magnitude of the zeta potential explains the lack of duration time dependence on voltage. The zeta potential of RNase A was also measured in buffers of different pHs. At pH 4 which is much lower than the pI 9.5, a positive zeta potential was obtained as expected. Also at a pH higher than the pI a negative zeta potential was obtained. This result highlights the importance of considering the zeta potential when performing nanopore analysis of proteins since nanopore experiments are usually performed with solutions of high salinity. Furthermore, while for simple peptides the effect of voltage on blockade time and current is consistent with a simple electrophoretic model of translocation, for proteins, there are clearly other parameters involved. Thus, a definitive answer to the question of protein translocation through the α-hemolysin pore cannot be obtained for all proteins through the indirect approach.

**Table 4:** RNase A zeta potentials in various buffers and pHs.

Buffer (pH)	Zeta Potential (mV)
10 mM KPi (pH 7.0)	−0.9±0.1
100 mM KCl, 10 mM KPi (pH 7.0)	−1.6±0.9
500 mM KCl, 10 mM KPi (pH 7.0)	−8.0±1.4
10 mM TRIS-HCl (pH 8.0)	−0.7±0.1
50 mM KCl, 10 mM TRIS-HCl (pH 8.0)	−1.8±0.3
50 mM KCl, 10 mM Sodium Citrate (pH 4)	13.2±0.9
50 mM KCl, 10 mM Sodium Carbonate/Bicarbonate (pH 10.0)	−13.3±2.5

### RT-PCR Detection of RNase A

As a result of the failed attempt to determine if RNase A translocates the α-hemolysin pore by applying an indirect approach, a direct approach was taken. Since RNase A is a robust protein and can readily refold to an active confirmation even if it unfolds to translocate the pore, it was reasoned that the presence of RNase A in the *trans* chamber solution (opposite from where the protein is initially added) can be detected using an activity based assay. RNase A is responsible for cleaving single-stranded RNA, thus one method for testing for RNase A activity is to employ the RT-PCR technique. RT-PCR technique is ideal in this case because it provides the sensitivity required. For example, α-hemolysin pore on average remains viable for only 2–5 hours, thus if a protein does indeed translocate only a few thousand molecules will go through the pore within that time frame. Therefore, a highly sensitive technique, such as RT-PCR is required.

**Figure 6:**
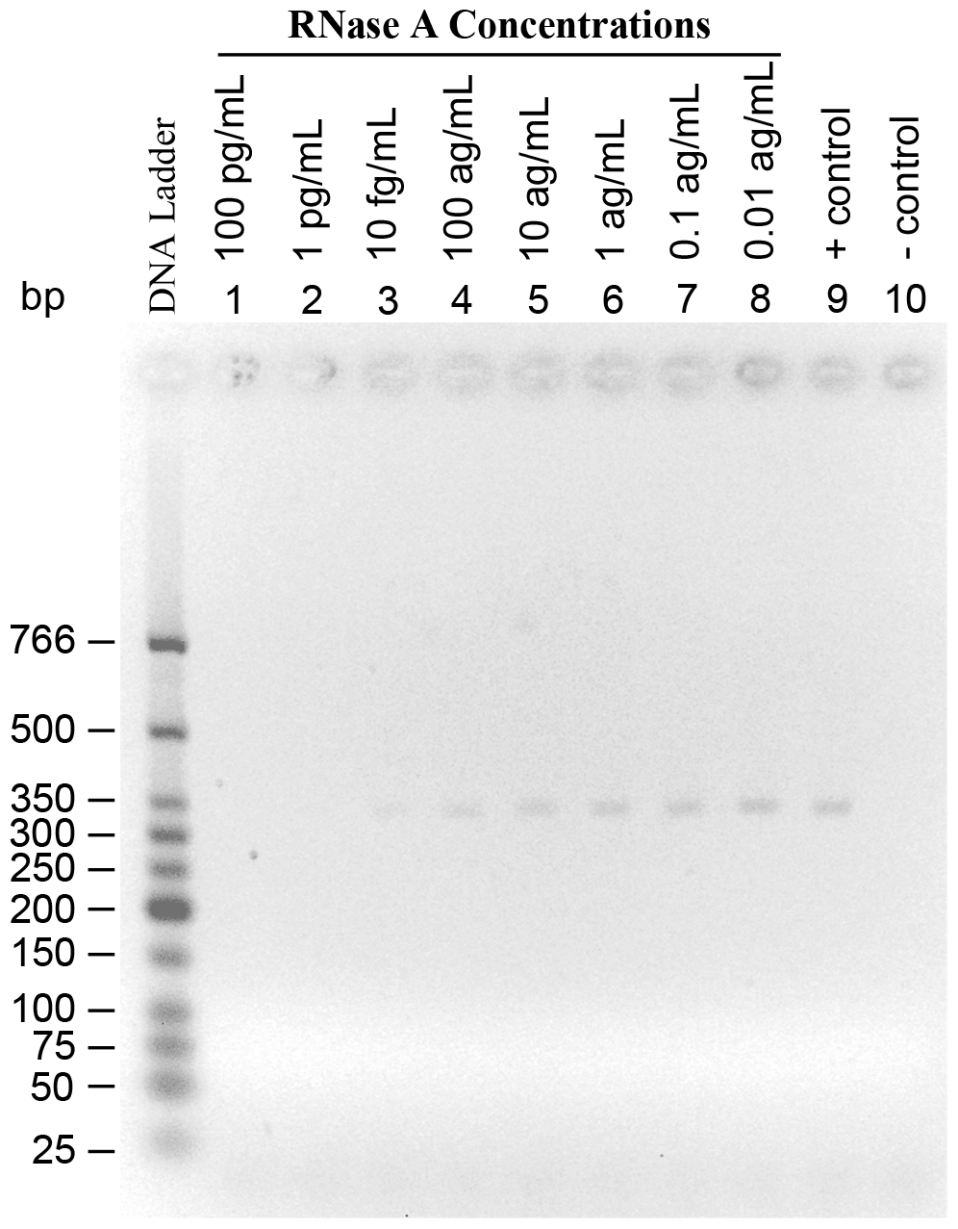
The detection limit of the RT-PCR based detection assay for RNase A. Lanes 1 to 8 indicate the concentrations of RNase A. Lanes 9 is the positive control for RT-PCR which contains no RNase A. The negative control for RT-PCR, lane 10, contains no RNase A or mRNA. The concentration of mRNA is the same in lanes 1 through 9.

We designed an RT-PCR based detection assay where RNase A is introduced into the *cis* chamber and a nanopore experiment is carried out. Thus, if translocation of the enzyme through the α-hemolysin pore is successful then some enzyme molecules will be present in the *trans* chamber. The solution of the *trans* chamber is collected and mRNA will be added to it. Upon incubating RNase A with the mRNA it will result in degradation of the mRNA which in turn will result in inhibition of the reverse transcription step of RT-PCR [57], [58]. As shown in Figure 6, this assay can detect at least 2200 molecules or 50 attograms of RNase A. If events with 60% or higher blockade amplitudes are assumed to be putative translocations, then recording 2200 events with blockade currents of 60% or higher is feasible. Hence, our RT-PCR based assay can be applied as a direct approach in determining if RNase A translocates α-hemolysin pore.

**Figure 7:**
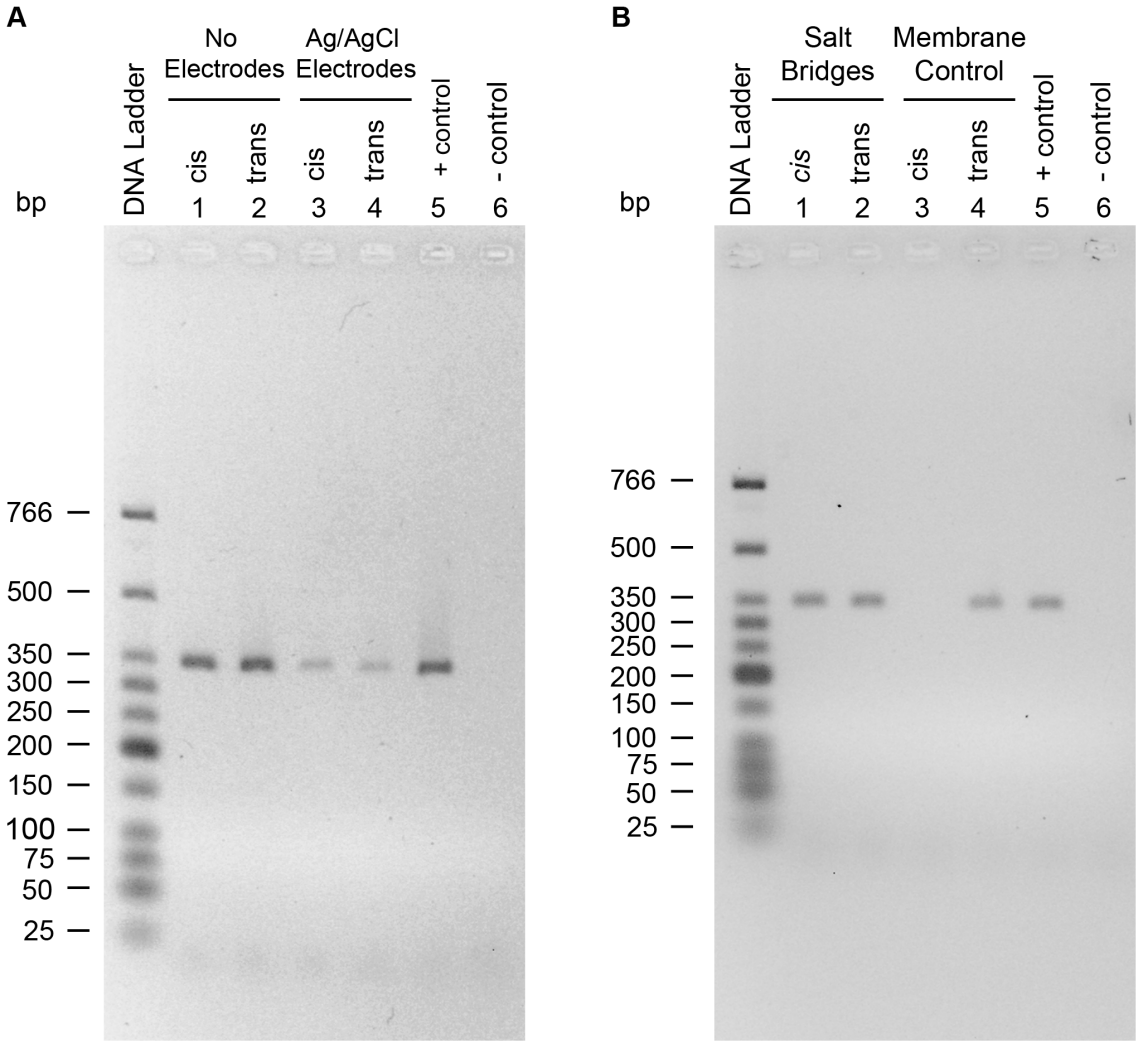
Effect of Ag/AgCl electrodes on the RNase A detection assay. (A) Lanes 3 and 4 show reverse transcription of mRNA when there are Ag/AgCl electrodes immersed in the solution, whereas lanes 1 and 2 show reverse transcription of mRNA when there are no electrodes immersed in solution. (B) Lanes 1 and 2 show reverse transcription of mRNA when there are agarose salt bridges immersed in the solution instead of Ag/AgCl electrodes. Lanes 3 and 4 show the *cis* and *trans* solutions, respectively, after adding RNase A to the *cis* chamber with the lipid bilayer membrane separating the two chambers.

As part of the process to ensure RNase A free apparatus, controls were run where both chambers, *cis* and *trans*, were filled with nuclease free electrolyte solution but there was no RNase A, lipid or α-hemolysin solution added to either chamber. This was done at the beginning of every experiment. Yet, after testing for RNase A activity there appeared to be some activity present in both chambers. Initially this was thought to be RNase A contamination but after a large number of control experiments it was found that this was due to the use of Ag/AgCl electrodes. This can be ruled out as RNase A contamination because the electrodes are stored overnight in bleach solution and they were pre-treated with an RNase A deactivating solution (RNase Zap, Applied Biosystems, Mississauga, Ontario) before running an experiment. So why is the use of Ag/AgCl electrodes resulting in false positive detection of RNase A? The simplest explanation is that there is silver leaching from the electrodes. The silver (I) leaching from the electrodes would bind to phosphate groups of the RNA backbone or to electron donor atoms on nucleobases [59], [60]. This binding forms ternary complexes between Ag (I) and nucleotides, cytosine and guanine [59], [60]. As a result of this binding, there would be a reduction in reverse-transcription of the mRNA. This hypothesis was tested directly by running control experiments where the *cis* and *trans* solutions were incubated with and without Ag/AgCl electrodes in absence of RNase A, lipid, and α-hemolysin. After 1 hr incubation period, the solutions were transferred to microcentrifuge tubes and the mRNA was added to each solution and then left for 24 hr incubation at 37°C. The mRNA from each solution was then used as a source of template RNA for RT-PCR. As shown in Figure 7A (lanes 1 and 2), in absence of electrodes there was successful reverse transcription of the mRNA (same intensity as the control lane, lane 5). In the presence of Ag/AgCl electrodes there is very little reverse-transcription of mRNA (Figure 7A, lanes 3 and 4). Upon this discovery agarose salt bridges were then used to avoid direct contact of the Ag/AgCl electrodes with the solutions of either *cis* or *trans* chambers. With the use of agarose salt bridges there is no effect on the reverse transcription of RT-PCR (Figure 7B, lanes 1 and 2). Figure 7B (lanes 3 and 4) shows another important control where addition of RNase A to the *cis* side results in no translocation to the *trans* side in the presence of a membrane even after few hours of incubation. Both of these findings are very important: the first finding shows the importance of salt bridges in nanopore analysis of nucleic acids and the latter finding confirms what is already expected; that is in absence of a pore there will be no protein translocation. The effect of silver leaching from the Ag/AgCl electrodes was also tested on nanopore analysis of proteins (data not shown). While there was some interaction between the silver leaching from Ag/AgCl electrodes and the protein, the effect on current blockade histogram profiles was very small. This could be because the amount of silver leaching from the electrodes might be extremely small compared to the amount of protein added (µg/mL). In the case of mRNA, the amount of mRNA (pg/mL) might be comparable to the amount of silver leaching from the electrodes. After the discovery of silver leaching from the Ag/AgCl electrodes, agarose salt bridges were used in all nanopore experiments with RNase A.

Nanopore experiments with RNase A were run and the number of events with current blocks of 60% or higher were recorded as putative translocations. Upon recording a high number of events with blockade currents of 60% or higher, the experiment was stopped and solution from both chambers were collected. The solution collected from each chamber was a fraction of the total volume in the chamber. This was done to avoid breaking of the membrane and in turn false translocation of the protein through the 150 µm aperture.

**Figure 8:**
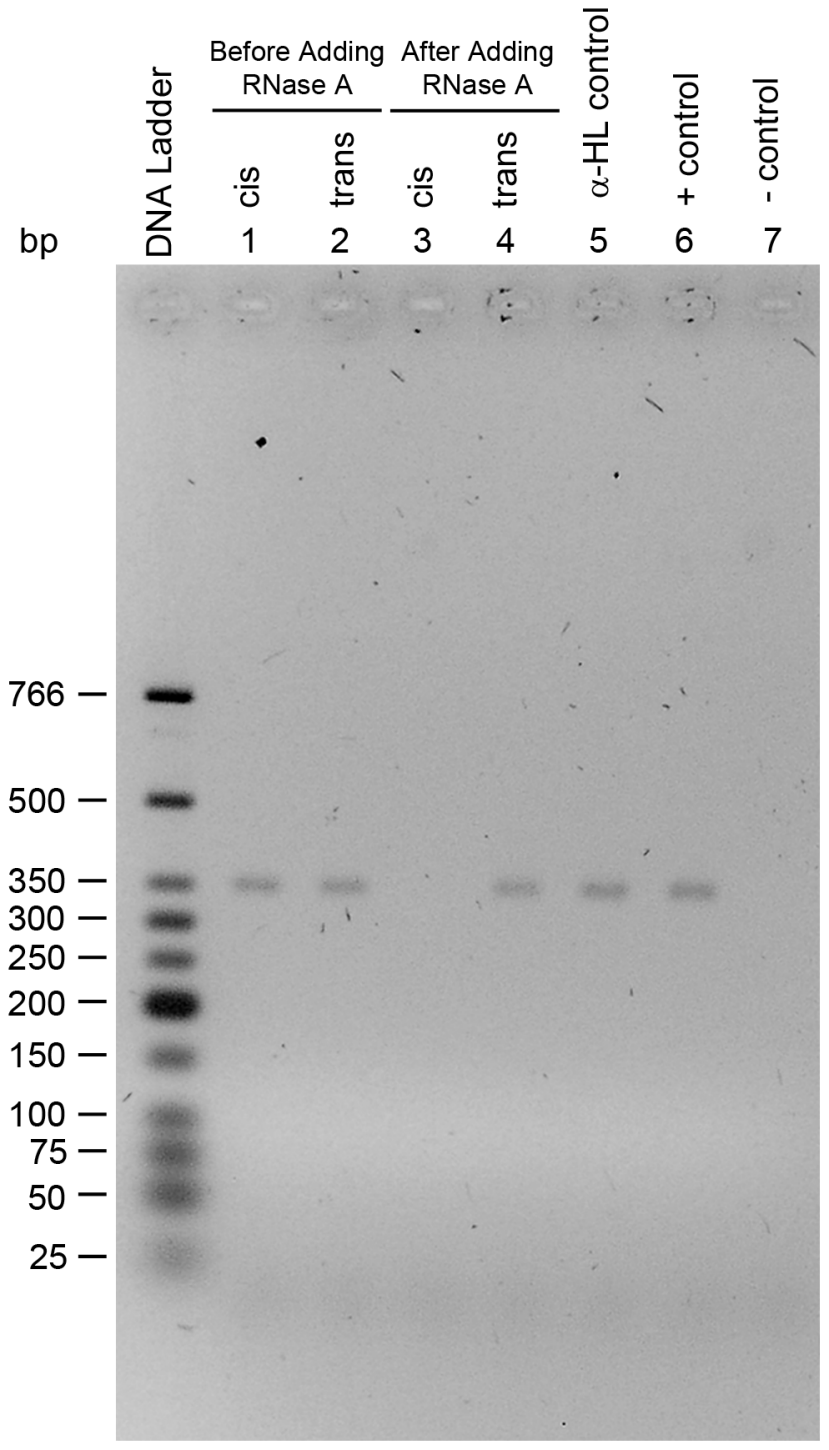
RT-PCR based detection of RNase A in the *trans* chamber. Lanes 1 and 2 represent the solutions collected from *cis* and *trans* chambers, respectively, before adding RNase A. Lanes 3 and 4 represent the solutions collected from cis and trans chambers, respectively, after adding RNase A to the cis chamber and conducting a nanopore experiment. The solutions used for lanes 3 and 4 were collected after the nanopore experiment and while the lipid bilayer separating the two chambers was still intact. Lane 5 represents a control for α-hemolysin solution used in the nanopore experiment where the α-hemolysin solution was tested for RNase A activity. Lanes 6 and 7 are positive and negative controls, respectively, for RT-PCR.

Once the nanopore experiment was completed, the RT-PCR based detection assay was used to test for activity in the solution collected from the *trans* chamber. As shown in Figure 8 (lane 4), there was no enzyme activity detected in the *trans* chamber. If there was any activity then the band in lane 4 would not be there or would be of lesser intensity than that of the positive control (lane 6). However, as expected there was RNase A activity in the *cis* chamber where the protein was added. Lanes 1 and 2 indicate controls where solutions were collected from both chambers prior to adding RNase A to the *cis* chamber. This was to ensure that the buffer and apparatus used are RNase A free. These experiments have been repeated numerous times (i.e more than 10 times). Therefore, the results shown in Figure 8 indicate that the large blockade events observed with RNase A are intercalation rather than translocation. This is a very important result because it is the first report of direct evidence which shows that large proteins don’t translocate the α-hemolysin pore.

**Figure 9:**
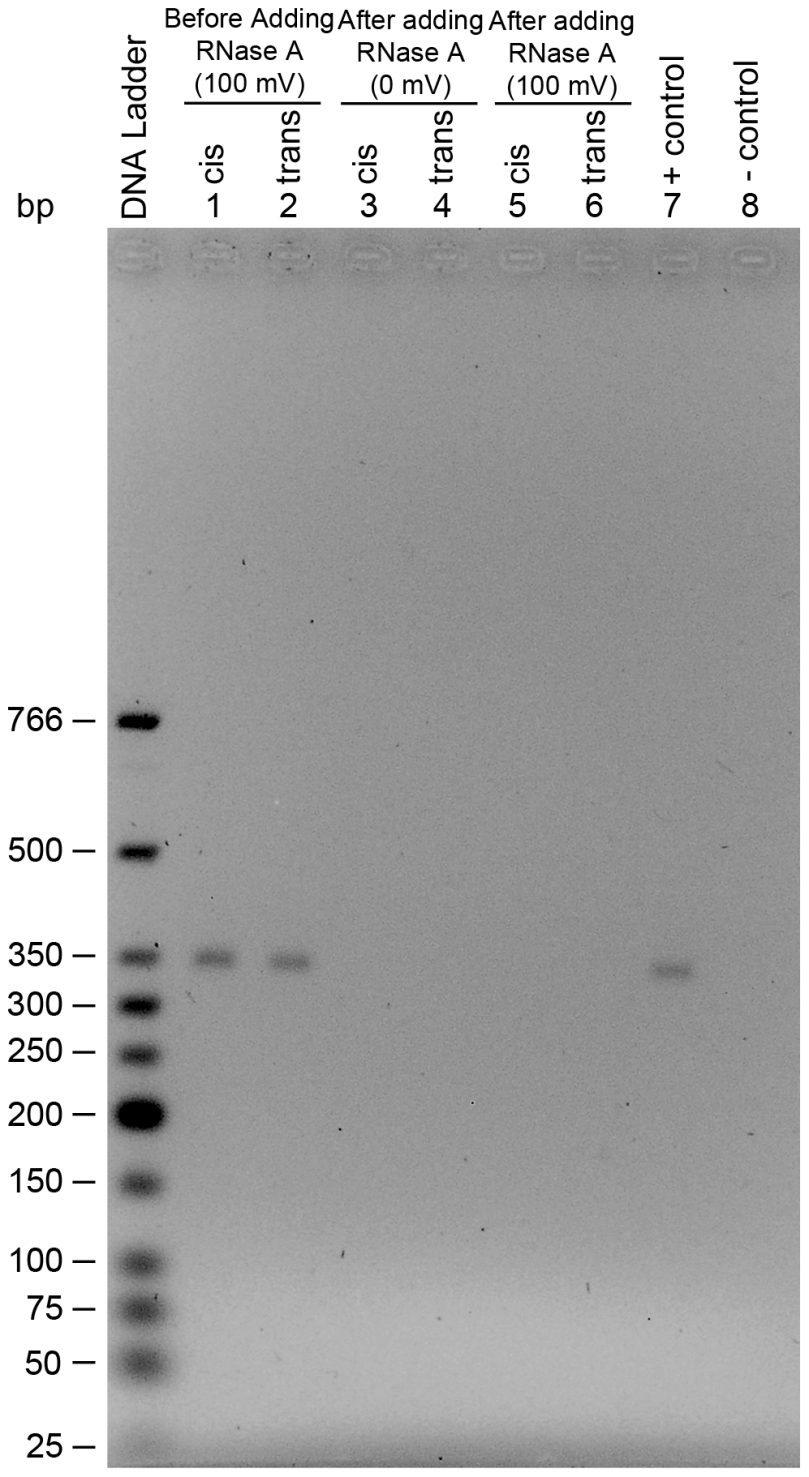
Translocation of RNase A through the 150 µm aperture. Lanes 1 and 2 are the solutions collected from the *cis* and *trans* chambers, respectively, before adding RNase A and while applying a potential of 100 mV. Lanes 3 and 4 are the solutions collected from cis and *trans* chambers, respectively, after adding RNase A to the *cis* chamber and under no applied voltage. Lanes 5 and 6 are similar to 3 and 4, respectively, but there was 100 mV applied. There was no lipid bilayer painted over the 150 µm aperture. Lanes 6 and 7 are positive and negative controls, respectively, for RT-PCR.

To determine if the size is indeed the limiting factor for RNase A translocation through the α-hemolysin pore, positive control experiments were run where the protein was added to the *cis* side but there was no membrane or pore present. Therefore, if size was the limiting factor then the protein could easily pass through the 150 µm aperture joining the two chambers. Indeed, as shown in Figure 9 (lane 4) the protein readily passes through the 150 µm aperture even in the absence of applied voltage; thus, indicating that protein translocation can be diffusion controlled. The same experiment was repeated when applying 100 mV (*cis* side grounded) and the protein translocated again (Figure 9, lane 6). These control experiments confirm that the size of the pore is the limiting factor for large protein translocation. Furthermore, this shows that if the pore is large enough then protein translocation can be diffusion controlled.

Similar experiments were attempted with completely unfolded ribonuclease A, it was impossible to conduct a nanopore experiment for sufficient period of time (i.e more than 3 hours) to record high number of events with 60% block or higher. This was as a result of couple of issues. First, because of the presence of denaturing and reducing agents (final concentrations of 240 mM and 6 mM, respectively), the membrane was less stable as compared to experiments conducted in the absence of denaturing and reducing agents. Thus, as a result it would break after a short period of time. While this might be acceptable in a typical nanopore experiment, this is not the case here because once the membrane breaks a new experiment must be restarted in order to prevent false positive translocations through the 150 µM aperture present in the cup. Second, after only few minutes of adding the unfolded RNase A to the *cis* chamber, the α-hemolysin pores would permanently block,possibly due to partly unfolded molecules. This in turn made it impossible to record sufficient number of large blockade events (i.e putative translocations). Finally, the third issue encountered was the frequency of events observed with the unfolded protein. Unexpectedly,the unfolded protein induced far fewer events than the folded protein. In an attempt to increase the frequency of events, the experiment was repeated in the presence of 1 M GdnHCl (*cis* chamber only). Interestingly, the presence of GdnHCl in both chambers improves the frequency dramatically but the presence of GdnHCl in *cis* chamber alone does not. However, having GdnHCl in the *trans* chamber will produce a false negative result on the RNase A detection assay. This is because even if the unfolded protein translocated the pore it will not be able to fold back into an active confirmation in the presence of denaturing agent. Hence, as a result of the limitations outlined here, a detection assay could not be carried out to determine if the unfolded RNase A translocates the α-hemolysin pore.

## Conclusions

The interaction of RNase A protein with the α-hemolysin pore in the absence or presence of a denaturing agent and/or reducing agent induces a large number of blockade events with large blockade currents. While the calculated net charge of the protein at pH 7 is positive, the zeta potential measurements, in similar condition to the ones used for nanopore analysis, reveals a charge reversal. The charge reversal indicates that under the experimental conditions used, the applied electrophoretic force will facilitate the translocation of the protein. In an attempt to determine if the electrophoretic force acting on the protein is sufficient in unfolding RNase A and subsequently aiding the translocation of the protein through the α-hemolysin pore, two different approaches were taken: an indirect approach and a direct approach.

In the indirect approach, the effect of voltage on the interaction of RNase A protein with the α-hemolysin pore was investigated. While the frequency of events increased with increasing voltage, the duration time remained unchanged independent of the applied voltage. Thus, the indirect approach failed to provide a definitive answer to protein translocation. In the direct approach, an RT-PCR based assay was used to test for RNase A activity in the *trans* chamber. The detection assay showed no RNase A activity even after running a nanopore experiment for 13 hours. This is the first report where a direct approach is applied to determine if a protein translocates the α-hemolysin pore. Furthermore, by applying the direct approach it's evident that the size of the pore is the limiting factor. With larger pores, the translocation of the protein can occur in the absence of applied electric field, that is by diffusion.
